# Corroboration and efficacy of Magneto-Fluorescent (NiZnFe/CdS) Nanostructures Prepared using Differently Processed Core

**DOI:** 10.1038/s41598-019-51631-w

**Published:** 2019-10-22

**Authors:** Dipti Rawat, P. B. Barman, Ragini Raj Singh

**Affiliations:** Department of Physics and Materials Science, Jaypee University of Information technology, Waknaghat Solan, 173234 H.P. India

**Keywords:** Nanoparticles, Synthesis and processing

## Abstract

The selected and controlled preparation of core@shell nanostructures, which unite the multiple functions of ferromagnetic Ni-Zn ferrite core and CdS shell in a single material with tuneable fluorescence and magnetic properties, have been proposed by the seed mediated aqueous growth process. The shell particle thickness and core of nanostructures were precisely tuned. Current work exhibits the comparative study of core@shell multifunctional nanostructures where core being annealed at two different temperatures. The core@shell nanostructure formation was confirmed by complementary structural, elemental, optical, magnetic and IR measurements. Optical and magnetic characterizations were performed to study elaborative effects of different structural combinations of core@shell nanostructures to achieve best configuration with high-luminescence and magnetic outcomes. The interface of magnetic/nonmagnetic NiZnFe_2_O_4_/CdS nanostructures was inspected. Unexpectedly, in some of the core@shell nanostructures presence of substantial exchange-bias was observed in spite of the non-magnetic nature of CdS QDs which is clearly an “optically-active” and “magnetically-inactive” material. Presence of “exchange-bias” was confirmed by the change in “magnetic-anisotropy” as well as shift in susceptibility derivative. Finally, successful formulation of stable and efficient core@shell nanostructures achieved, which shows no exchange-bias and shift. Current findings suggest that these magneto-fluorescent nanostructures can be used in spintronics; and drug delivery-diagnosis-imaging applications in nanomedicine field.

## Introduction

Nanostructures are found to be of great magnitude for the reason that of their vital properties, such as, large surface/volume ratio and the engineered characteristic’s such as, permeability, stability, and porosity^1^. Core@shell nanostructures have been a dynamic area of research for their tailored multifunctional properties arising as a result of the existence of two or more nanostructure phases of different materials with one forming the shell on the surface of the other^2–7^. In the current work we have studied the core@shell nanostructure of NiZnFe_2_O_4_/CdS, which is a ferromagnetic/diamagnetic core@shell system. The core material chosen was NiZnFe_2_O_4_ which is a soft ferrite. The reason to opt soft ferrite is there properties such as high resistivity which means “low eddy current loss” and “high usable frequency” ranges, ‘high magnetic permeability” which means high induction in smallest space, adaptability of shape of the core which persuade magnetic requirements in nominal space and their light-weight and low-cost relative to other materials. In our core@shell system the well-controlled interface between the two components give rise to exciting properties due to the presence of two different components at the surface. This ferromagnetic/diamagnetic system of core@shell nanostructures has been studied using various methods.

Magnetic properties of the core and the interface in core@shell nanostructure have been studied using vibrating sample magnetometry measurements (VSM) at room temperature. Optical studies were performed using UV-visible spectroscopy and Photoluminescence spectroscopy at room temperature. HRTEM images give the straightforward proof for the formation of core@shell nanostructure and the formation of sharp interface between two components. “The coherency strain, which allows the shell substantial at the interface to fine-tune the lattice parameters of the core, can play an important role in such core@shell nanostructure systems^8^”. These magneto-fluorescent nanostructures can be used in various applications such as spintronics; as-well-as for drug delivery-diagnosis-imaging applications in the field of nanomedicine^7,9–11^.

## Results and Discussions

### Structural analysis

**Figure 1:**
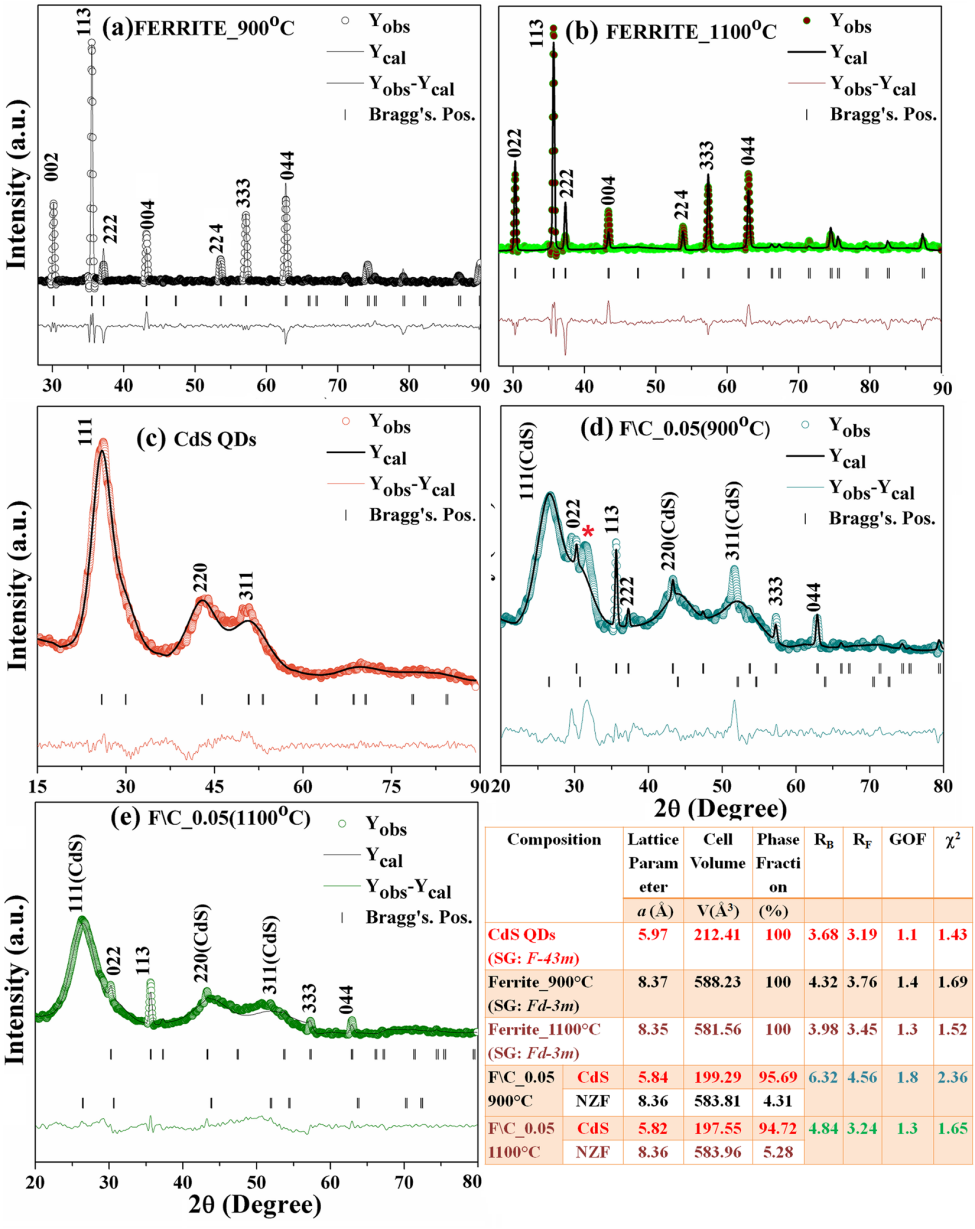
The Rietveld refinement pattern of X-ray diffraction spectra for the (**a**,**b**) NiZnFe_2_O_4_; annealed at different temperatures (**c**) CdS QDs; and (**d**,**e**) NiZnFe_2_O_4_/CdS core@shell nanostructures.

In the present study we have studied the X-ray diffraction spectrum for NiZnFe_2_O_4_ core, CdS QDs shell and core@shell nanostructures of NiZnFe_2_O_4_/CdS with core being annealed at two different temperature 900 °C and 1100 °C respectively as presented in Fig. 1(a–e). Peaks shown in XRD spectrum for NiZnFe_2_O_4_ annealed at 900 °C (Fig. 1(a)) and 1100 °C (Fig. 1(b)) have been indexed as (022), (113), (222), (004), (333), and (044) hkl planes indicating the formation of “single-phase spinel cubic structure” of nickel zinc ferrite (JCPDS No. 52-0277). The as-prepared nanoparticles do not exhibit any impurity phase. The intensity of the highest peak (113) is enhanced for the 1100 °C annealed NiZnFe_2_O_4_ nanoparticles as compared to 900 °C annealed NiZnFe_2_O_4_, showing better crystallinity. On increasing the annealing temperature of NiZnFe_2_O_4_ from 900 °C to 1100 °C the crystallite size was found to be decreased from 29.96 nm to 23.75 nm as calculated using Scherrer’s formula. This decrease in crystallite size with increase in annealing temperature is due to increased micro strain^12^.

In CdS spectra (Fig. 1(c)) the peaks are indexed as (111), (220) and (311) hkl planes representing the cubic structure of CdS QDs (JCPDS No. 80-0019) with crystallite size approx. 1.83 nm. Figure 1(d,e) shows the XRD spectra for the core@shell nanostructures with minimal NiZnFe_2_O_4_ loading of 0.05 g. Diffraction peaks corresponding to NiZnFe_2_O_4_/CdS exhibited peaks both from the CdS QDs and for the NiZnFe_2_O_4_. Figure 1(d) have an extra peak which is found to be of ZnS when confirmed through JCPDS card No. 05-0566. This trace of ZnS in case of core@shell nanostructure with 900 °C annealed NiZnFe_2_O_4_ is due to intermixing of Zn present in ferrite with the sulphur used in CdS QD’s synthesis. While in case of core@shell nanostructure with 1100 °C annealed NiZnFe_2_O_4_ (Fig. 1(e)) there is no extra peak. Thus, we can say that at higher NiZnFe_2_O_4_ annealing temperature the core@shell system is more intact. This is an affirmative point for core@shell formation, where, there is no diffusion of core material with the shell and vice-versa. This point is further confirmed through optical and EDX results. Shift in the peaks was observed for all core@shell nanostructures on comparing with bare core and separate shell structures which confirms the successful formation of core@shell nanostructures. All the nanostructures were found to contain pure crystalline phases deprived of any type of alloying in the samples. Particle size calculation for core@shell nanostructures was not achievable by XRD because these spectra include distinct peaks for NiZnFe_2_O_4_ and CdS QDs. From the literature we have got the same trend of XRD spectra for different type of magnetic core and semionductor shell type core-shell nanostructures^13–15^. [More structural data for prepared nanostructures have been presented in Table 1 of Supplementary Data].

W-H plot for the NiZnFe_2_O_4_, CdS QDs and the NiZnFe_2_O_4_/CdS core@shell nanostructures (Supplementary Data Fig. 1) was used to find out more structural parameters. The attained values of the crystallite size from W-H plot are comparable to those calculated using “Scherer’s’ formula. The crystallite size found to be 38.50 nm for ferrite annealed at 900 °C, 30.80 nm for the ferrite annealed at 1100 °C and 2.70 nm for CdS QDs. Strain estimated from Williamson-Hall (“W-H”) method are also inscribed in figures. The positive slope of linear fit in the supplementary data Fig. 1(a,c,g) shows the tensile strain present in the sample. 900 °C annealed NiZnFe_2_O_4_ ferrite system (Supplementary Data Fig. 1(b)) is having very low value of strain i.e., 0.001 whereas 1100 °C annealed NiZnFe_2_O_4_ has large micro-strain value of 2.62. CdS QDs posses 0.054 strain value, these results explain the effect of small particle size on higher strain values. In the core@shell nanostructures (Supplementary Data Fig. 1(d,e)) the negative slope is there and it indicates the compressive strain in the system, using this we can not calculate the particle size through W-H plot because the equation does not satisfied. Moreover, this result is one of the proof for core@shell formation. The negative slope of Supplementary Data Fig. 1(d,e) shows that macrostrains cannot be a leading source of broadening. Through these results it can be concluded that the effect of crystallite size and micro strains is negligible in the core@shell nanostructures.

The observed XRD spectra were refined by employing Rietveld refinement technique using the Full Prof software^16,17^. The corresponding calculated profiles (represented by solid black curve) and difference between observed and calculated profile (represented by the coloured line) have also been shown in Fig. 1. The lattice parameters (‘*a*’) obtained from Rietveld refinement in case of pure ferrite and in core@shell structure are in agreement with the experimental results and the values are close to the some of the previously reported values^18,19^. However, ‘*a*’ in case of pure CdS is found to be larger in comparison to core/shell structures this is because of the tensile stress present in pure CdS but as we grow CdS QDs on ferrite core there will be generation of compressive strain that gives the ‘*a*’ near to standard values. The experimental data (colour spectra) is well matched with the refined data points (black spectra) for the cubic CdS QDs, spinel cubic phase of NiZnFe_2_O_4_ and the two mixed phases of core@shell nanostructures, with the space group of *F*-*43m* for CdS QDs and *Fd*-*3m* for NiZnFe_2_O_4_. The value of goodness of fit for all the samples was found to be near 1 which indicates toward the good fitting of data points. Rietveld refinement analysis ruled out the presence of any type of impurity in the nanostructures except in core/shell nanostructure where core has been annealed at 900 °C, have an extra peak which is found to be of ZnS. Therefore the Rietveld parameters found weaker in this specific sample in comparison to other nanostructures specifically in comparison to other core@shell structure. In case of core@shell nanostructures mixed cubic faces of core and shell have been achieved. Summary of results obtained by Rietveld refinement analysis were collected in Fig. 1.

### Absorbance spectroscopy

**Figure 2:**
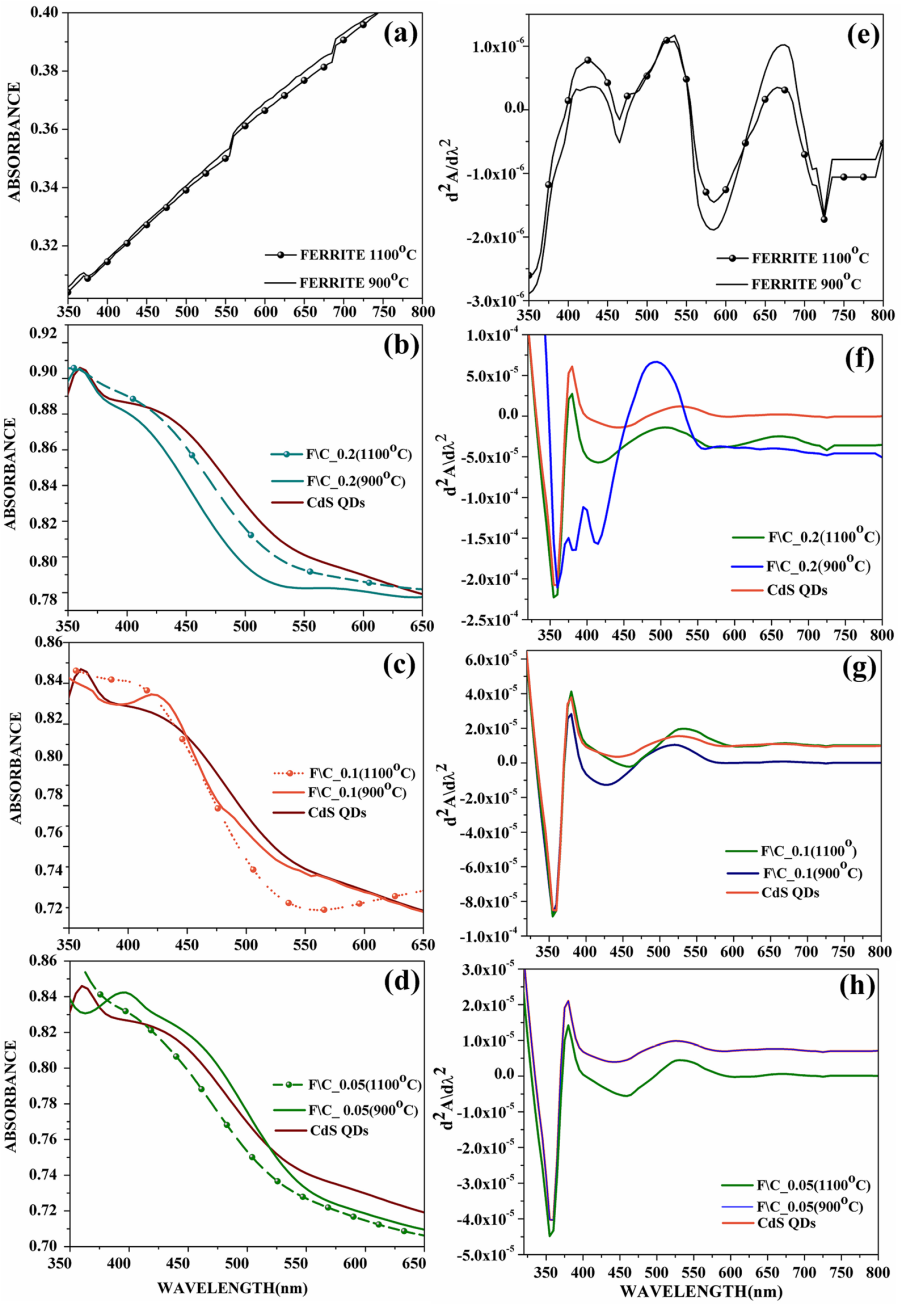
Absorbance spectra of (**a**) NiZnFe_2_O_4_; NiZnFe_2_O_4_/CdS nanostructures (**b**) with 0.2 g; (**c**) with 0.1 g; (**d**) with 0.05 g ferrite loading. Derivative absorbance spectra of (**e**) NiZnFe_2_O_4_; (**f**) NiZnFe_2_O_4_/CdS nanostructures with 0.2 g loading; (**g**) NiZnFe_2_O_4_/CdS nanostructures with 0.1 g loading; (**h**) NiZnFe_2_O_4_/CdS nanostructures with 0.05 g loading.

UV-visible spectrum for NiZnFe_2_O_4_, CdS QDs and their core@shell nanostructures have been recorded in order to study the optical behaviour of prepared structures (Fig. 2(a–d)) with different loadings of NiZnFe_2_O_4_. Figure 2(a) represents the absorbance spectra of NiZnFe_2_O_4_ prepared through annealing at 900 °C and 1100 °C and the graph clearly indicates no absorption behaviour in the range 350–800 nm, which is of course the range of interest in order to prepare magneto-fluorescent structures. CdS QDs exhibits the band edge absorption at 379.60 nm and can be seen in the Fig. 2(b–d). In the core@shell nanoparticles with varied NiZnFe_2_O_4_ loadings, slight shift in absorption edge maxima has been observed (Fig. 2(b–d)) in case where core was annealed at 900 °C. “The variation in absorption edge was due to the introduction of synergistic effects of low absorption ferrite nanoparticles along with the high absorption CdS QD^20^”. The maximum NiZnFe_2_O_4_ loading (0.2 g) shows a shift in absorption toward lower wavelength side in case of core@shell nanostructures with NiZnFe_2_O_4_ being annealed at 900 °C (Fig. 2(b)). Also, in case of core@shell nanostructures with NiZnFe_2_O_4_ being annealed at 1100 °C the absorption edge slightly shifts to the blue side of the spectrum in comparison with the position of CdS QDs absorbance.

In the core@shell nanostructures with varying NiZnFe_2_O_4_ loading the absorption maxima at distinct positions have been obtained [Table 2 of Supplementary Data] and absorption is being shifted toward the blue side of the spectrum which signifies about the decrease in particle size of CdS QDs on shell formation over NiZnFe_2_O_4_ core. Moreover, there is lattice mismatch in the NiZnFe_2_O_4_ and CdS QDs structures eventually it produces compressive strain on the system, specifically in the CdS QDs as vindicated by the W-H plots of the core@shell nanostructures. Moreover, it is well known that there is relation between the increased strain in the system to the energy band gap and eventually to the particle size of the QDs^21^. Therefore, the obtained absorbance spectra of core@shell nanostructures clearly showcased the effect of increased strain via blue shifted absorbance profiles.

Derivative spectroscopy (DS) uses either first or higher derivative of absorbance with reference to wavelength for qualitative analysis and quantification. “The impression of derivative spectra data was presented first in the 1950s, when it was revealed to have many advantages^22–24^”. If a spectrum is presented as absorbance “A”, as a function of wavelength, “λ”, the derivative spectra will be:

$${0}^{{\rm{th}}}\,{\rm{order}},\,{\rm{A}}={\rm{f}}({\rm{\lambda }});\,{{\rm{I}}}^{{\rm{st}}}\,{\rm{order}},\,\mathrm{dA}/d{\rm{\lambda }}={\rm{f}}^{\prime} ({\rm{\lambda }});\,{{\rm{II}}}^{{\rm{nd}}}\,{\rm{order}},\,{{\rm{d}}}^{2}{A/d{\rm{\lambda }}}^{2}={\rm{f}}^{\prime\prime} ({\rm{\lambda }})$$

I^st^ order derivative is calculated as the rate of change of absorbance with respect to wavelength it begins and ends at zero. The main trait of the even-order derivatives is a “strong negative” or “positive band” with maximum or minimum at the same wavelength as λ_max_, of the absorbance band. An important point to note is that the numbers of bands observed in spectra are always equal to the derivative order plus one. Figure 2(e–h) shows the derivative of absorbance for the respective nanostructures. By using the second order derivative of absorbance for the respective samples we have calculated the exact absorbance positions for samples (Table 2 Supplementary Data). Figure 2(e–h) shows the second order derivative for the bare NiZnFe_2_O_4_, CdS QDs and their core@shell nanostructures. In the double derivative graphs three peaks are clearly seen in each case, which is the clear indication of second order derivation where the bands observed are equal to derivative order plus one. In case of NiZnFe_2_O_4_ Fig. 2(e) the three bands at almost same position have been observed. In case of core@shell nanostructures with 0.2 g NiZnFe_2_O_4_ loading (Fig. 2(f)) some extra peaks are observed. Derivative spectra with core annealed at 900 °C as compared to; pure CdS and core@shell nanostructure with NiZnFe_2_O_4_ annealed at 1100 °C these extra peaks are due to some alloying of NiZnFe_2_O_4_ with CdS shell material and this band is due to ZnS formation during core@shell formation which has already been discussed in XRD results. Core@shell nanostructures with NiZnFe_2_O_4_ annealed at 1100 °C give no such indication of alloying and give the peaks almost at the same position as obtained in CdS QDs. This shows that the core became more intact as the annealing temperature of core has been raised. In case of core@shell nanostructures with 0.1 g loading (Fig. 2(g)) some traces of intermixing are found in case of core@shell formed of 900 °C annealed NiZnFe_2_O_4_ as can be seen from shifting in the peaks. This alloying is completely eliminated in case of core@shell nanostructures with minimal NiZnFe_2_O_4_ loading (0.05 g) (Fig. 2(h)) where all the CdS QDs, NiZnFe_2_O_4_/CdS (900 °C) and NiZnFe_2_O_4_/CdS (1100 °C) bands positioned similarly (no shifting) and also presenting no presence of extra peak.

### Photoluminescence study

**Figure 3:**
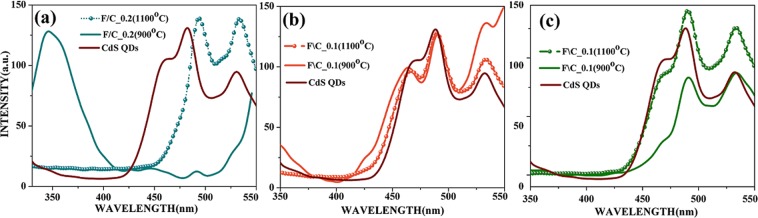
Photoluminescence spectra of (**a**) NiZnFe_2_O_4_/CdS nanostructures with 0.2 g loading; (**b**) NiZnFe_2_O_4_/CdS nanostructures with 0.1 g loading; (**c**) NiZnFe_2_O_4_/CdS nanostructures with 0.05 g loading with respect to bare CdS QDs.

**Figure 4:**
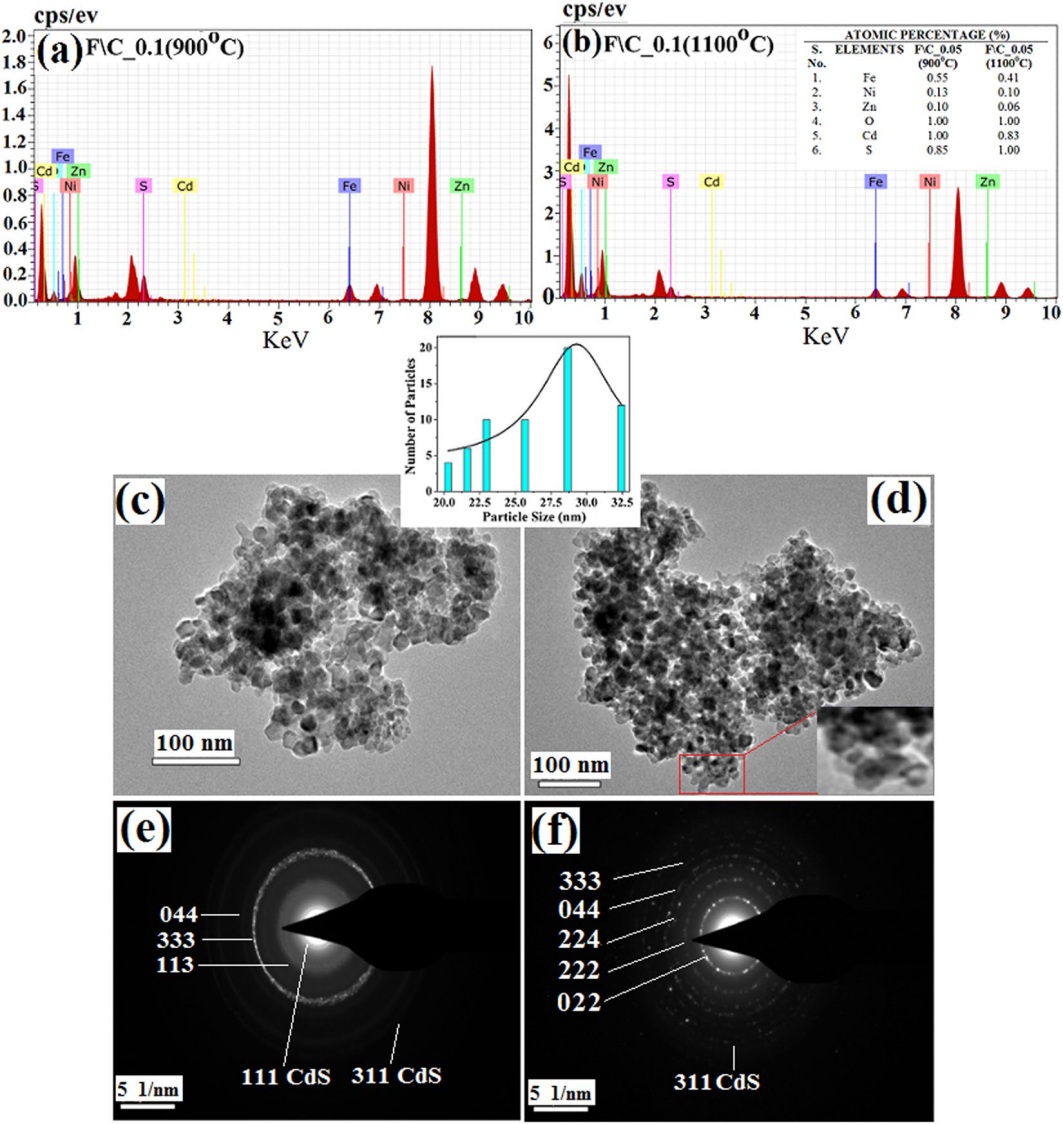
EDX spectra of core@shell nanostructures with (**a**) 0.05 g loading of NiZnFe_2_O_4_ (900 °C); and (**b**) 0.05 g loading of NiZnFe_2_O_4_ (1100 °C) with CdS2. TEM micrograph of core@shell nanostructures with (**c**) 0.05 g loading of NiZnFe_2_O_4_ (900 °C); and (**d**) 0.05 g loading of NiZnFe_2_O_4_ (1100 °C) with their respective SAD patterns (**e**,**f**).

Room temperature photoluminescence spectra of CdS QDs along with different core@shell nanostructures of NiZnFe_2_O_4_/CdS were recorded by dispersing the 0.02 mg/ml sample in the water and are shown in Fig. 3. The PL spectra of entire NiZnFe_2_O_4_/CdS2 core@shell nanostructures and CdS2 QDs have been recorded with the excitation wavelength of 290 nm. Corresponding emission wavelengths and intensities of the peaks observed in PL for all the nanostructures have been summarized in Table 1. Fluorescence spectra of CdS QDs were observed with intense emission band as compared to their core@shell nanostructure which includes magnetic nanoparticles. A high intensity band was obtained at 482.2 nm which was due to de-trapped charge carrier recombination. A weak band at 530.5 nm, attributed to recombination of trapped electrons and holes present at the surface defect sites was also observed. However, the PL dynamics of CdS QDs is distinctly different from that of the NiZnFe_2_O_4_/CdS nanostructures with different loading of NiZnFe_2_O_4_ core, in case of core@shell nanostructures with 0.2 g ferrite (annealed at 900 °C) loading (Fig. 3(a)) a very intense peak at 345 nm was found and matched with ZnS. The ZnS peak may be due to the presence of high amount of Zn present in NiZnFe_2_O_4_ to react with sulphur present in thiourea in the process of CdS QDs synthesis. This ZnS peak is also evident from the XRD analysis of core@shell nanostructures. This clearly indicates that NiZnFe_2_O_4_ annealed at 900 °C is not very much stable as it releases the zinc present in ferrite to mix with sulphur of CdS. Thus, the NiZnFe_2_O_4_ annealed at 1100 °C is much stable as compared to 900 °C annealed NiZnFe_2_O_4_ as there is no leakage of Zn from NiZnFe_2_O_4_ to CdS QDs shell. While in case of NiZnFe_2_O_4_ loading of 0.1 g (Fig. 3(b)) and 0.05 g (Fig. 3(c)) the ZnS peak is not present, better luminescent intensity and the better particle size distribution in case of minimal NiZnFe_2_O_4_ loading has been achieved (Fig. 4(c)). Therefore, this minimal ferrite loading has been opted out for further studies throughout the manuscript.

**Table 1:** Summary of peak positions and intensities of CdS QDs and NiZnFe_2_O_4_/CdS nanostructures prepared with 0.2 g, 0.1 g and 0.05 g loading of NiZnFe_2_O_4._

S. No.	Sample name	Peak positions (nm)			Intensities (a.u)		
		E_1_	E_2_	E_3_	I_1_	I_2_	I_3_
1.	CdS QDs	460.2	482.3	530.5	106.7	130.0	125.0
2.	F\C_0.2 (900 °C)	345.6	491.0	—	128.9	20.1	—
3.	F\C_0.2 (1100 °C)	494.1	532.7	—	137.1	136.1	—
4.	F\C_0.1 (900 °C)	463.6	489.7	533.0	91.2	122.3	130.4
5.	F\C_0.1 (1100 °C)	464.0	489.0	533.9	90.0	122.3	98.5
6.	F\C_0.05 (900 °C)	466.7	490.4	533.5	33.1	83.4	87.9
7.	F\C_0.05 (1100 °C)	466.7	490.4	533.5	86.2	136.5	126.6

### Energy-dispersive X-ray spectra and TEM

We have calculated the relative concentration of elements present in the EDX spectra. The EDX spectra of core@shell nanostructures (900 °C and 110 °C) are being presented in (Fig. 4(a,b)). The stoichiometry ratios of prepared samples have been measured by EDX for elemental compositions in core@shell nanostructures with NiZnFe_2_O_4_ (900 °C) and of NiZnFe_2_O_4_ (1100 °C) with minimal ferrite loadings. The results revealed that the core@shell nanostructure prepared with 900 °C (Fig. 4(a)) annealed NiZnFe_2_O_4_ is off-stoichiometry, while prepared with 1100 °C (Fig. 4(b)) annealed NiZnFe_2_O_4_ all the elements are found to be in expected compositional ratio. Availability of excess oxygen is due to the presence of oxygen in the formula of compound and in environment during characterization. The decrease in sulphur content in case of 900 °C annealed ferrite core as compared to 1100 °C is due to the formation of ZnS in case of 900 °C annealed core, which has also been evidenced from the XRD results and the optical study results.

HR-TEM analysis has been used to analyse the shape and size of the synthesized core@shell nanostructures. Investigation of the core@shell nanostructures is done with samples containing minimal NiZnFe_2_O_4_ loading in the cases where core being annealed at 900 °C and 1100 °C. Figure 4(c,d) shows the HRTEM images of core@shell nanostructures. Before examination, the samples were ultrasonicated for 20 min in ethanol to avoid agglomeration. From Fig. 4(c,d) it is clearly seen that there are sharp interfaces in case of core@shell nanostructure prepared with 1100 °C annealed NiZnFe_2_O_4_, as compared to 900 °C annealed NiZnFe_2_O_4_ core. These sharp interfaces are due to the stability of core at high temperatures, which restricts the intermixing at the interface and we can see the clear black coloured core surrounded by the grey colour shell. Inset shows the zoomed image of core@shell nanostructures. The particle size distribution of core/shell nanostructures is also shown in Fig. 4 and it is in good agreement of XRD data of core and shell samples separately, and confirms the size of core/shell structures approximately 29 nm but it is not possible to find out the particle size from XRD results. HRTEM images also supports the structural and optical results where we have talked about the stability of core@shell nanostructures at higher annealing temperature. Figure 4(e,f) shows the indexed SAD pattern for nanostructures. These figures show the selected area electron diffraction pattern for the core@shell nanostructures with NiZnFe_2_O_4_ being annealed at two different temperatures. Both the patterns are showing the ring formation made up of small spots, every spot occurred from Bragg’s reflection from a single crystallite which is the clear indication of poly-nanocrystalline nature of core@shell nanostructures.

### Magnetic characterization

**Figure 5:**
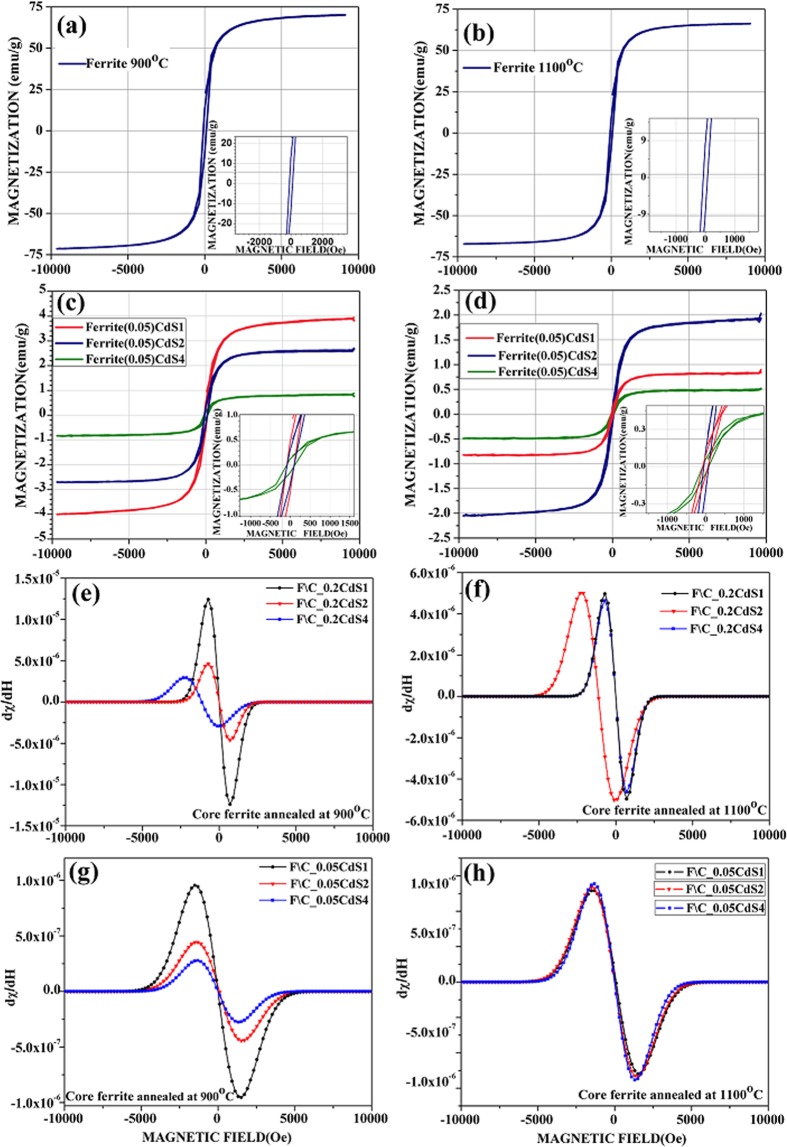
Hysteresis loops for NiZnFe_2_O_4_ (**a**) 900 °C, (**b**) 1100 °C; and NiZnFe_2_O_4_/CdS with NiZnFe_2_O_4_ (**c**) 900 °C, (**d**) 1100 °C; at room temperature with different particle sized shell. dχ/dH curves of NiZnFe_2_O_4_/CdS nanostructures with varying CdS QDs layers with 0.2 g NiZnFe_2_O_4_ loading (**e**) 900 °C, (**f**) 1100 °C; and with 0.05 g NiZnFe_2_O_4_ loading (g) 900 °C, (**h**) 1100 °C.

Magnetization measurements of NiZnFe_2_O_4_ annealed at two different temperatures 900 °C and 1100 °C and NiZnFe_2_O_4_/CdS core@shell nanoparticles have been measured. The magnetic hysteresis loop for core and core@shell nanostructures is illustrated in Fig. 5(a–d), there was no need to perform the VSM analysis of shell CdS QDs as these are purely diamagnetic in nature. The measured values for saturation magnetization (M_s_), retentivity (M_r_), coercivity (H_c_),) magnetic anisotropy (K) and the magnetic moment (μ_B_) are listed in Table 2. The saturation magnetization (M_s_) of NiZnFe_2_O_4_ core annealed at two different temperatures 900 °C (Fig. 5(a)) and 1100 °C (Fig. 5(a)) was found to be 70.16 emu/g and 66.35 emu/g respectively. The decrease in magnetization with increased annealing temperature is due to the excessive temperature which accelerates the ion-diffusion and formation of pores during annealing. As a result, the domain wall movement was not easy and may be the cause of decreased magnetization. Due to development of pores at higher annealing temperatures samples have the decrease in saturation magnetization. Moreover, samples experience an induced spin-reorientation transition, by a temperature change and reduced by an external magnetic field^25^.

**Table 2:** Magnetic parameters for NiZnFe_2_O_4_ and core@shell nanostructures.

S. No.	Sample	M_s_ (emu/g)	M_r_ (emu/g)	H_c_ (Oe)	K (erg/cm^3^)	M_μ_ (μB)
1.	FERRITE_900 °C	70.16	13.04	98.98	3472.12	2.97
2.	FERRITE_1100 °C	66.35	6.45	73.16	2427.02	2.81
3.	F\C_0.05CdS1 (900 °C)	3.91	0.44	91.00	177.90	0.27
4.	F\C_0.05CdS1 (1100 °C)	1.96	0.14	64.00	62.72	0.13
5.	F\C_0.05CdS2 (900 °C)	2.65	0.33	84.5	111.96	0.18
6.	F\C_0.05CdS2 (1100 °C)	0.81	0.08	69.00	27.94	0.06
7.	F\C_0.05CdS4 (900 °C)	0.82	0.12	98.50	40.38	0.06
8.	F\C_0.05CdS4 (1100 °C)	0.48	0.045	82.45	19.78	0.03

In case of core@shell nanostructures with shell of CdS1, CdS2 and CdS4 at the room temperature the saturation magnetization was found to be in the range of 2.65 emu/g~0.48 emu/g respectively (Fig. 5(c,d)). The saturation magnetization was decaying from pure ferrites to core@shell nanostructures; this is because of the non-magnetic CdS QDs shell formation over the NiZnFe_2_O_4_core^26^. Moreover, the phase fraction of ferrites (core) and CdS QDs (shell) from Rietveld analysis shows the reduced fraction of core ferrite and this is in the coordination with decay in saturation magnetization with core@shell formation and is another proof of the core@shell formation. In spite of the decrease in magnetic nature of core@shell in NiZnFe_2_O_4_/CdS, the well preserved coercivity in the core@shell nanostructure is an excellent sign for their bio-applications^26^. From the Table 2 it can be noted that there is the decreasing trend of anisotropy as we switch from pure NiZnFe_2_O_4_ to NiZnFe_2_O_4_/CdS core@shell nanostructure this indicates about the intact core surface even after CdS QDs shell formation as if there is any increase in anisotropy it could result from the alloying of core@shell interface and can introduce adverse changes in the magnetic as well as optical properties of core@shell nanostructures. Moreover, Magnetic moment is calculated using the formula, μB = (M_W_ × M_S_)/5585, where M_w_ is the molecular weight of compound and, M_S_ is the magnetic saturation of respective sample. Variation of magnetic moment is tabulated in Table 2 and indicates the direct relation with magnetic saturation^27^. Moreover, the core@shell nanostructures prepared using NiZnFe_2_O_4_ core annealed at 1100 °C which is the higher temperature is more stable and demonstrates the better suitability in terms of physical and chemical stability as seed material and hence possessing low anisotropy values. The coercivity values tend to decrease on increasing core ferrite loading, while the anisotropy values are decreasing with the increase of NiZnFe_2_O_4_ loading in the core@shell nanostructures prepared using NiZnFe_2_O_4_ (1100 °C) as core with varying CdS QD layers.

From the graphs Fig. 5(e–h) it is clearly seen that with decreasing the particle size of shell as CdS1, CdS2 and CdS4 (where 1 ml, 2 ml and 4 ml concentration of 2-mercaptoethenol has been used as the surfactant to controlled growth of shell particles) the magnetic susceptibility and the magnetic saturation are decreasing. This decrease is related to the surface to volume ratio of the shell CdS QDs. As the particle is becoming small the band gap is increasing and hence the conductivity is decreasing and the shell starts becoming more diamagnetic. Therefore, the susceptibility is least in case of core@shell nanoparticles with shell formed of CdS4 as compared to other CdS QDs shells.

By solving the derivative of susceptibility, we can comment about the spin interaction of core@shell at the interface. From peak width we can get the information about the spin interactions. In case of core@shell nanostructures with maximum ferrite loading the peak position and the peak width are changing, in case of 900 °C (Fig. 5(e)) annealed core NiZnFe_2_O_4_ there are major changes as compared to the 1100 °C (Fig. 5(f)) annealed NiZnFe_2_O_4_. Since we are only altering the particle size of shell keeping the ferrite loading fix, the change in peak position is due to the effect of shell on the core system. There may be some spin-spin interaction at the interface. In case of core@shell nanostructures with minimal NiZnFe_2_O_4_ loading of 0.05 g, there is very negligible shift in both the set of samples (Fig. 5(g,h)). In the core@shell system of 900 °C annealed core, there is negligible shift in peak position, but the susceptibility is decaying as the particle size of shell is decreasing. While in case of core@shell nanostructures with NiZnFe_2_O_4_ being annealed at 1100 °C both the peak position and the susceptibility are well maintained. Thus, we can say that prepared core@shell system is progressing towards more intactness and stability at higher core annealing temperature and minimal core loading. Therefore, prepared sample can find many applications in biological field where best properties are needed at the cost of minimum amount of sample.

### FTIR analysis

**Figure 6:**
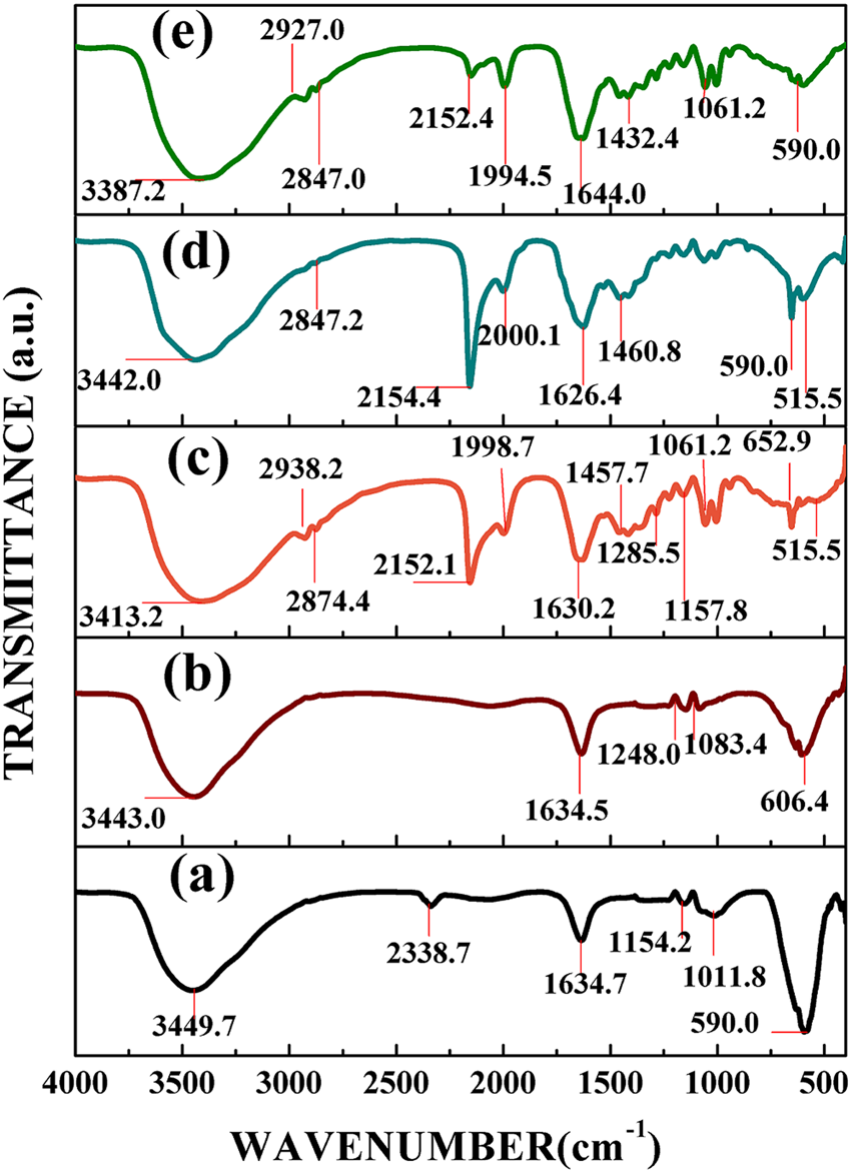
FTIR spectra of NiZnFe_2_O_4_ annealed at two different temperatures (**a**) 900 °C; (**b**) 1100 °C; (**c**) CdS QDs; and the core@shell nanostructures of NiZnFe_2_O_4_/CdS QDs prepared using ferrite annealed at two different temperatures (**d**) NiZnFe_2_O_4_ (900 °C)/CdS; and (**e**) NiZnFe_2_O_4_ (1100 °C)/CdS.

FTIR investigation was directed to recognize unknown materials as well as the number of components and the quality or consistency of our samples. Figure 6 shows the FT-IR spectra for nickel-zinc ferrite annealed at two different temperatures, CdS QDs, and the core@shell nanostructures of NiZnFe_2_O_4_@CdS. Ferrites, 2-mercaptoethanol capped CdS QDs and core@shell nanostructures were inspected through FTIR spectra in the range of 4000–400 cm^−1^. Figure 6(a,b) shows the FT-IR spectra for pure ferrite annealed at 900 °C and 1100 °C respectively. In general, in the FTIR spectra of spinel ferrites two main broad oxygen metal bands have been observed which are also present in our samples. Therefore, the one, observed around ν_1_ = 606 cm^−1^ corresponds to inherent stretching vibration of the metal ion at the “tetrahedral site”, whereas the lowest band, that observed around ν_2_ = 460 cm^−1^ is assigned to “octahedral” metal stretching vibration^28^. The band appearing near about at 3388 cm^−1^ corresponds to O–H stretching vibration of H_2_O; the special absorption peak at 2339 cm^−1^ corresponds to O–H group of citric acid; the band at 1450 cm^−1^ was due to antisymmetric NO_3_ stretching vibrations and the band at 1647cm^−1^ was due to carbo-oxalate anions^29–32^. The stretching vibration at 2352 cm^−1^ corresponds to the hydroxyl group. In Fig. 6(c) the broad peak at 2120 cm^−1^, 1383 cm^−1^ and the weak peak at 1614 cm^−1^ were assigned to C=C, C–H characteristic vibrations in the CdS QDs samples. The band appearing near about at 3418 cm^−1^ corresponds to O−H stretching vibration of H_2_O, the stretching vibration at 2352 cm^−1^ corresponds to the hydroxyl group.

These bands correspond to the mixture of the solution formed by metal nitrates and citric acid. The most attractive part of FTIR spectra, with respect to NiZn Ferrites, is in the range 800–250 cm^−1^. This range is dispensed to the ion vibrations in the crystal lattice. The ν_1_ band found between 800 and 500 cm^−1^, which is due to the stretching-vibrations occurred inside the tetrahedral sites. Another absorption ν_2_, is present in the middle of 450 and 300 cm^−1^. This band has been assigned to stretching-vibrations inside the octahedral sites. For all samples in Fig. 6, the analysis point out absorption peaks near about at 530 cm^−1^ corresponds to the Fe–O vibration associated to the magnetite phase^33^. The results of the significant shift of 1634 cm^−1^ to 1630 cm^−1^ lower frequency indicates that the nanoparticles were in a closed-packed, crystalline state. In case of core@shell nanostructures (Fig. 6(d,e)) at some points we get the mixed peaks of both the ferrite and the CdS QDs.

## Conclusion

- Demonstrated that the core@shell nanostructures of NiZnFe_2_O_4_/CdS can be synthesized successfully by seed mediate growth method having combination of sol-gel and solution growth methods.
- Structural analysis with the help of Rietveld refinement and; morphological characterizations depict the well-defined phases and high crystalline quality of NiZnFe_2_O_4_ core and CdS QDs shell.
- HR-TEM images and SAD pattern also confirms the core@shell nanostructure formation.
- CdS QDs shell was successfully employed upon the exterior of the Ni-Zn ferrite nanoparticles and a clear addition was observed in all the cases especially in minimal ferrite loading core@shell nanostructure.
- Optical studies discovered that the obtained nanostructures are stable and emit high intensity fluorescence and the best results were obtained at the minimal loading of core material.
- VSM investigation showed the formation of ferromagnetic NiZnFe_2_O_4_/CdS nanostructures.
- The spin dependent studies are performed on NiZnFe_2_O_4_/CdS nanostructures using derivative of susceptibility. In case of maximum amount of core loading and at lower annealing temperature of core ferrite it was found that the core@shell system formed has diffused boundaries, the spin-spin interaction of both the core and shell take place at the interface. While in case of minimal amount of core loading and at higher annealing temperature of core, core@shell system found to be more chemically and physically stable. Therefore, both core and shell are intact. Magnetic study also reveals that with semiconductor shell formation over magnetic core, saturation magnetization decays which is the clear indication of shell formation over core.
- FTIR studies divulged the functional groups present on the system are favourable to further process these magneto-fluorescent nanostructures for biomedical applications.
- Overall, findings suggest that these magneto-fluorescent nanostructures can be used in spintronics; as-well-as for drug delivery-diagnosis-imaging applications in the field of nanomedicine.

## Methods

### Production of NiZnFe_2_O_4_/CdS core@shell nanostructures

We have employed the “seed growth method” to synthesise the core@shell nanostructure where NiZnFe_2_O_4_ was the core material upon which CdS QDs shell was grown using solution growth technique. Two different sets of core@shell nanostructure samples have been prepared by varying the annealing temperature of core, core loading and the particle size of CdS QDs shell formed over ferrite core. For the NiZnFe_2_O_4_ synthesis, we have mixed the stoichiometric ratios of nitrates according to their adjusted composition in the distilled water and the continuous stirring is applied until the nitrate solution completely dissolved in distilled water to form the solution of metal nitrates precursors. Metal nitrates precursors were then mixed in aqueous solution of citric acid monohydrates and the nitrate to citric acid molar ratio and maintained to 1:3. pH of the solution was adjusted to 7 by using ammonia solution. The reaction mixture was then heated at 75 °C ± 5 °C with continuous stirring for several hours till the liquid solution changes to the viscous gel. The obtained gel was then dried at 110 °C for 22 h in the hot air oven. Dried powder was then annealed at 900 °C and 1100 °C respectively for five hours to form NiZnFe_2_O_4_ nanoparticles. Henceforth, in direction of preparing NiZnFe_2_O_4_/CdS core@shell nanostructures, the as prepared NiZnFe_2_O_4_ nanoparticles annealed at two different temperatures were added as core (seeds) during the CdS QDs synthesis. The shell formation reaction which was employed in our synthesis mechanism consists of CdCl_2_, NH_4_Cl, and thiourea in molar ratio of 1:1.5:3. Firstly, salts of ammonium chloride and cadmium chloride were added in 50 ml of double distilled water with continuous stirring and heating. After that as the temperature reaches 70 °C ± 5 °C, ammonia solution was added drop wise to maintain the pH of solution 7.5, next as prepared NiZnFe_2_O_4_ was added and immediately after that thiourea and 2-mercaptoethanol were added. The reaction was continued for 3 hours with constant heating and stirring. Thereafter samples were cleaned and collected for further characterization. In the first set of samples we have synthesised the core@shell nanostructures with core annealed at 900 °C and in the second set of samples core annealed at 1100 °C. The core loading was 0.02 g, 0.1 g and 0.05 g and the different sized particle shell formed using 2-mercaptoethanol solution in different amount as CdS1, CdS2, and CdS4 are the CdS QDs prepared by adding 1 ml, 2 ml and 4 ml of 5% 2-mercaptoethanol solution in the process of CdS growth.

### Characterization methods

X-ray diffraction studies have been carried out using “Shimadzu 6000 diffractometer” equipped with Cu-K_α_ radiation within the scan range from 10°–80° with a scan speed of 2 degree/minute, operated at “40 kV and 30 mA”. Rietvel refinement has been performed to study the crystal structure using FULLPROF A. Absorption spectra have been recorded in the wavelength range 200 nm to 800 nm, using “Perkin Elmer Lambda 750 spectrophotometer”. “LS-55 spectrophotometer (Perkin Elmer)” has been used to record “photoluminescence (PL) “spectra in the range 350–550 nm at an excitation-wavelength of 290 nm at room temperature. Magnetic measurements have been obtained using “PAR-155 vibrating sample magnetometer (VSM)” in the range “−10 KOe to +10 KOe” at room temperature. High resolution transmission electron microscope (HRTEM) and Energy Dispersive Spectroscopy (EDS) were carried by using “FP 5022/22-Tecnai G2 20 S-TWIN model”. FT-IR analysis is done through “RX-IFTIR Perkin Elmer”.

## Supplementary information

Supplemetary Data

## Data Availability

The datasets generated during and/or analysed during the current study are available from the corresponding author on reasonable request.
