# Supplementary material for: Corroboration and efficacy of Magneto-Fluorescent (NiZnFe/CdS) Nanostructures Prepared using Differently Processed Core
Source: Sci Rep. 2019 Oct 22;9:15138. doi: 10.1038/s41598-019-51631-w (PMC6805930; doi:10.1038/s41598-019-51631-w)

**Corroboration and efficacy of Magneto-Fluorescent (NiZnFe/CdS) Nanostructures Prepared using Differently Processed Core**

**Dipti Rawat, P.B. Barman and Ragini Raj Singh^*^**

*Department of Physics and Materials Science, Jaypee University of Information technology, Waknaghat Solan-173234, H.P., India*

*^*^Corresponding author:* [*raginirajsingh@gmail.com*](mailto:raginirajsingh@gmail.com)

**Table 1.** Structural parameters for NiZnFe_2_O_4_ , CdS QDs and NiZnFe_2_O_4_/CdS QDs core@shell nanostructures.

| **Sample name** | **2θ (deg)** | **d(Å)** | **FWHM (deg)** | **Intensity** | **hkl** | **Particle size**  **(D)nm** | **Particle size (D)nm (W-H plot)** | **Lattice constant (Å)** |
| --- | --- | --- | --- | --- | --- | --- | --- | --- |
| **Ni-Zn ferrite**  **900 ^o^C** | 30.18 | 2.96 | 0.28 | 2905 | 022 | 29.96 | 38.50 | a=8.34 |
|  | 35.53 | 2.52 | 0.31 | 5030 | 113 |  |  | a=8.35 |
|  | 37.17 | 2.48 | 0.3 | 2091 | 222 |  |  | a=8.11 |
|  | 43.18 | 2.09 | 0.32 | 2498 | 004 |  |  | a=8.36 |
|  | 53.59 | 1.70 | 0.31 | 2170 | 224 |  |  | a=8.32 |
|  | 57.12 | 1.63 | 0.34 | 2734 | 333 |  |  | a=8.36 |
|  | 62.73 | 1.47 | 0.36 | 2931 | 044 |  |  | a=8.31 |
| **Ni-Zn ferrite**  **1100 ^o^C** | 30.24 | 2.95 | 0.34 | 1584.75 | 022 | 23.75 | 30.80 | a=8.34 |
|  | 35.67 | 2.51 | 0.35 | 2601.53 | 113 |  |  | a=8.32 |
|  | 37.22 | 2.41 | 0.37 | 1166.78 | 222 |  |  | a=8.34 |
|  | 43.18 | 2.09 | 0.35 | 1333.97 | 004 |  |  | a=8.36 |
|  | 53.81 | 1.70 | 0.37 | 1231.04 | 224 |  |  | a=8.32 |
|  | 57.18 | 1.60 | 0.37 | 1514.24 | 333 |  |  | a=8.31 |
|  | 63.13 | 1.47 | 0.41 | 1610.91 | 044 |  |  | a=8.31 |
| **CdS2** | 26.85 | 3.31 | 3.98 | 329.39 | 111 | 1.83 | 2.70 | a=5.73 |
|  | 44.29 | 2.04 | 5.25 | 146.71 | 220 |  |  | a=5.76 |
|  | 51.01 | 1.78 | 4.75 | 135.17 | 311 |  |  | a=5.90 |
| **Ni-Zn Ferrite (0.05) CdS2 900 ^o^C** | 26.84 | 3.31 | 3.84 | 377.25 | 111 (CdS) | - | - | - |
|  | 31.68 | 2.82 | 1.84 | 201.62 | 022 |  |  |  |
|  | 35.66 | 2.51 | 0.34 | 176.86 | 113 |  |  |  |
|  | 43.53 | 2.07 | 2.49 | 184.36 | 004 |  |  |  |
|  | 51.69 | 1.79 | 1.19 | 169.52 | 311 (CdS) |  |  |  |
|  | 57.34 | 1.63 | 0.48 | 112.59 | 333 |  |  |  |
|  | 62.94 | 1.48 | 0.45 | 115.11 | 044 |  |  |  |
| **Ni-Zn Ferrite (0.05) CdS2 1100 ^o^C** | 26.51 | 3.35 | 3.76 | 423 | 111 (CdS) | - | - | - |
|  | 30.19 | 2.95 | 1.28 | 241 | 022 |  |  |  |
|  | 35.80 | 2.50 | 0.32 | 247 | 113 |  |  |  |
|  | 43.27 | 2.08 | 2.15 | 224 | 004 |  |  |  |
|  | 50.73 | 1.75 | 0.36 | 194 | 311 (CdS) |  |  |  |
|  | 57.26 | 1.60 | 0.39 | 144 | 333 |  |  |  |

**Table 2:** Absorption edge calculated using second order derivative of absorbance spectra.

| **S.No.** | **SAMPLE NAME** | **ABSORBANCE POSITION** | | |
| --- | --- | --- | --- | --- |
|  |  | **A_1_** | **A_2_** | **A_3_** |
| **1.** | CdS | 379.60 | 527.29 | 653.41 |
| **2.** | F\C_0.2 CdS(900^0^C) | 374..87 | 395.29 | 495.17 |
| **3.** | F\C_0.2 CdS(1100^0^C) | 379.63 | 527.29 | 653.41 |
| **4.** | F\C_0.1 CdS(900^0^C) | 379.86 | 517.62 | 651.52 |
| **5.** | F\C_0.1 CdS(1100^0^C) | 376.60 | 527.29 | 653.41 |
| **6.** | F\C_0.05 CdS(900^0^C) | 379.60 | 527.29 | 653.41 |
| **7.** | F\C_0.05 CdS(1100^0^C) | 376.03 | 533.99 | 652.04 |

**Figure 1. WH plot for Ferrites, CdS quantum dots and Core/Shell nanostructures.**


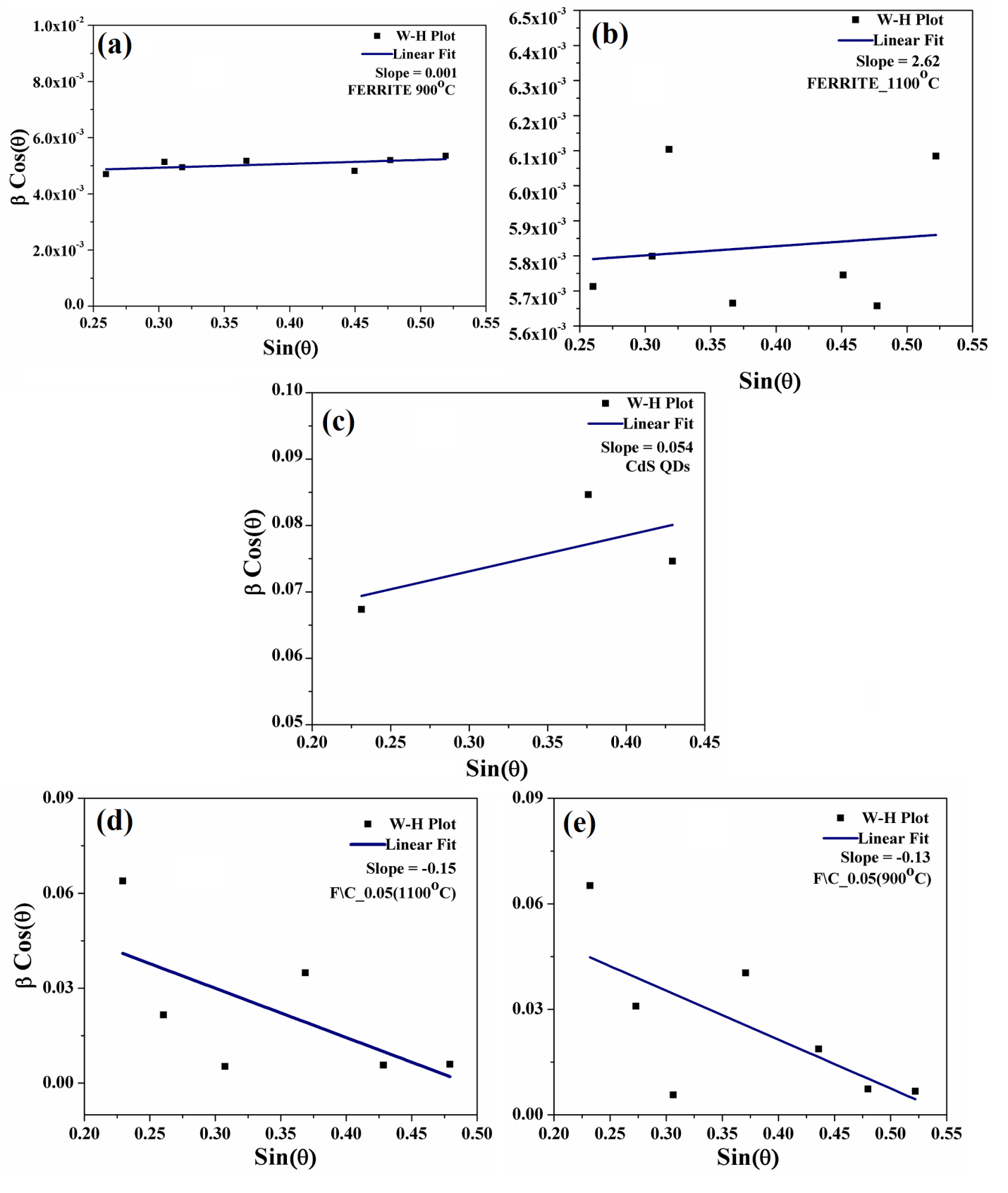

Supplement: Supplementary file 1 — Supplemetary Data [file 41598_2019_51631_MOESM1_ESM.docx]
